# Placenta Previa et Percreta: A Potentially Life-Threatening Condition

**DOI:** 10.3390/diagnostics13030539

**Published:** 2023-02-01

**Authors:** Cornelia Bachmann, Harald Abele, Markus Hoopmann

**Affiliations:** Department for Women's Health, University Hospital Tübingen, 72076 Tübingen, Germany

**Keywords:** placenta previa, percreta, caesarean section, life-threatening condition, pregnancy, placenta accreta spectrum, ultrasound, scar infiltration, diagnosis, imaging

## Abstract

Placenta percreta occurs in about 5% of placenta accreta spectrum (PAS) and causes high maternal and fetal peripartum morbidity/mortality. A 34-year-old multiparous 4G2P (1xcesarean section (CS)) was admitted to hospital at the 34th week of gestation. Transvaginal ultrasound revealed a placenta previa totalis et percreta with a small tissue layer towards the bladder. Ultrasound was crucial for further planning. An interdisciplinary setting was established based on this life-threatening diagnosis. Due to the onset of labor one day later, a CS was performed. Intraoperatively, the suspicion was confirmed of a placenta previa et percreta with CS scar infiltration. Due to the life-threatening bleeding risk, simultaneous subtotal hysterectomy was needed. The diagnosis was confirmed histologically. The higher the number of previous CS, the higher the PASrate. Placenta percreta is the most severe form of this, characterized by placental invasion through the entirety of the myometrium and possibly into extrauterine tissues. This case demonstrates the great importance of prenatal diagnosis with the realization of dimensions of this very rare finding, especially with an increasing CS rate and other associated complications. Due to the close interdisciplinary cooperation of the prenatal diagnosticians, obstetricians, and anesthesiologists with optimal care in a specialized center, the otherwise high morbidity/mortality can be minimized.

**Figure 1:**
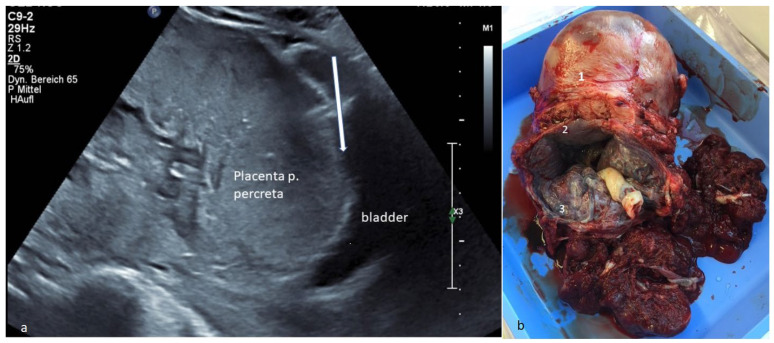
(**a**) Transvaginal ultrasound: presentation of a placenta previa totalis et percreta at 34 weeks gestation with a small tissue layer (arrow) towards the urinary bladder. Placenta percreta occurs in about 5% of placenta accreta spectrum (PAS) [1,2] and causes high maternal and fetal peripartum morbidity/mortality. Ultrasound was crucial for further planning. A eutrophic fetus with regular Doppler is present. (**b**) Specimen: The specimen of the subtotal resected uterus is demonstrated. The uterus (1) is marked, and the placenta previa totalis et percreta (3) detected preoperatively on ultrasound in the lower uterine segment (CS scar (2)) is clearly seen. Intraoperatively, the suspicion was confirmed with a placenta previa et percreta with CS scar infiltration. Due to the life-threatening bleeding risk, simultaneous subtotal hysterectomy was needed. Placenta percreta is the most severe form, characterized by placental invasion through the entirety of the myometrium and possibly into extrauterine tissues [2]. The diagnosis was confirmed histologically. Maternal blood loss was 1.7 L (2 red-blood-cell concentrates were administered). There was a good maternal and fetal outcome (fet 2490 g, inconspicuous APGAR/pH). The most common predisposing risk factor is a previous CS [3,4], as in the case presented. The higher the number of previous CS, the higher the PASrate [2]. Typically, PAS is associated with placenta previa [3] as well. Sometimes, life-threatening hemorrhage can occur, which often requires a blood transfusion [2,5]. The gold standard treatment is cesarean hysterectomy [2]. This case demonstrates the great importance of prenatal diagnosis with immediate realization of the dimensions of this very rare finding, especially with the increasing CS rate and other associated complications [4,6,7].

## Data Availability

All data regarding this case are published within it, for further questions please ask the authors.
